# Amidoxime Group‐Anchored Single Cobalt Atoms for Anti‐Biofouling during Uranium Extraction from Seawater

**DOI:** 10.1002/advs.202105008

**Published:** 2022-01-22

**Authors:** Wenyan Sun, Lijuan Feng, Jiacheng Zhang, Ke Lin, Hui Wang, Bingjie Yan, Tiantian Feng, Meng Cao, Tao Liu, Yihui Yuan, Ning Wang

**Affiliations:** ^1^ State Key Laboratory of Marine Resource Utilization in South China Sea Hainan University Haikou 570228 P. R. China

**Keywords:** amidoxime, anti‐biofouling, reactive oxygen species, single atoms, uranium extraction

## Abstract

Marine biofouling is one of the most significant challenges hindering practical uranium extraction from seawater. Single atoms have been widely used in catalytic applications because of their remarkable redox property, implying that the single atom is highly capable of catalyzing the generation of reactive oxygen species (ROS) and acts as an anti‐biofouling substance for controlling biofouling. In this study, the Co single atom loaded polyacrylamidoxime (PAO) material, PAO‐Co, is fabricated based on the binding ability of the amidoxime group to uranyl and cobalt ions. Nitrogen and oxygen atoms from the amidoxime group stabilize the Co single atom. The fabricated PAO‐Co exhibits a broad range of antimicrobial activity against diverse marine microorganisms by producing ROS, with an inhibition rate up to 93.4%. The present study is the first to apply the single atom for controlling biofouling. The adsorbent achieves an ultrahigh uranium adsorption capacity of 9.7 mg g^−1^ in biofouling‐containing natural seawater, which decreased only by 11% compared with that in biofouling‐removed natural seawater. These findings indicate that applying single atoms would be a promising strategy for designing biofouling‐resistant adsorbents for uranium extraction from seawater.

## Graphical abstract

Based on the ability of the amidoxime group to bind both uranyl and Co^2+^ ions, the Co single‐atom‐loaded PAO‐Co material is fabricated for uranium extraction and anti‐biofouling. The PAO‐Co material exhibits a broad range of antimicrobial activity against marine biofouling by producing reactive oxygen species and a high uranium extraction capacity of 9.7 mg g^−1^ in biofouling‐containing natural seawater.

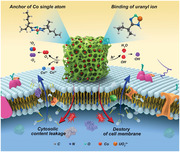

## 1 Introduction

Monatomic materials theoretically possess 100% atom utilization efficiency, unique activity, and selectivity, which endows them great potential for rationally using metal resources in diverse areas.^[^ ^1^ ^]^ Isolated metal atoms are more active than nano or sub‐nano metal particles due to their low coordination, unsaturated atoms as active sites, and quantum size effect.^[^ ^2^ ^]^ However, single atoms are easily reactive and agglomerate owing to their high surface free energy and mobility. Therefore, isolated atoms need to be anchored on various substrates to form stable configurations.^[^ ^3^ ^]^ According to previous reports, covalent or ionic interactions with neighboring atoms stabilize isolated metal atoms on solid frameworks. The strong interaction between single atoms and carriers makes their electronic structure tunable, empowering the single atom with unique properties for many essential applications,^[^ ^2^, ^4^ ^]^ including electrocatalytic oxygen reduction,^[^ ^5^ ^]^ oxygen evolution,^[^ ^6^ ^]^ hydrogen evolution,^[^ ^2^, ^7^ ^]^ carbon dioxide reduction,^[^ ^8^ ^]^ and nitrogen reduction.^[^ ^9^ ^]^ These applications are based on the remarkable redox properties of single atoms, implying that the single atom has a high potential to produce reactive oxygen species (ROS) by the oxidation reactive. ROS are highly active chemical substances, and their main types are free radicals, including singlet oxygen (^1^O_2_), superoxide anion radicals (·O_2_^−^), and highly active hydroxyl radicals (·OH).^[^ ^10^ ^]^ Because of their high oxidation activity, ROS are found to work as antimicrobial substances by destroying organic components, including proteins and DNA, of biological cells.^[^ ^11^ ^]^ However, to date, there is rare reports on the application of single atoms in the production of ROS and antimicrobial activity of single atoms.

With the rapid development of society, environmental security, and energy accessibility are of great significance for human survival and development.^[^ ^12^ ^]^ Nuclear energy is a clean energy source that can generate abundant energy without generating greenhouse gases harmful to the environment.^[^ ^13^ ^]^ Uranium is a crucial element in the nuclear‐energy industry. However, uranium resources on land are limited, and there is an urgent need to explore new supplies of uranium resources.^[^ ^11^, ^14^ ^]^ Seawater contains thousands of times more uranium resources than land reserves, and the extraction of uranium from seawater is considered the most promising approach to meet the growing demands of uranium.^[^ ^15^ ^]^ Numerous adsorbents have been fabricated for extracting uranium from seawater, and many of them exhibit promising uranium extraction capacity in natural seawater under laboratory conditions.^[^ ^16^ ^]^ However, seawater is highly complex, possessing high ion strength and complicated interfering ions that hinder the extraction of uranium in deficient concentrations. Additionally, seawater contains abundant biofouling of marine microorganisms and other biological entities, limiting the practical application of uranium adsorbents in natural ocean environments.^[^ ^17^ ^]^ Because of the diversity of potentially polluting organisms, it is challenging to create antifouling materials capable of extracting uranium from marine environments. Currently, several anti‐biofouling uranium adsorbents have been designed for marine biofouling. However, a large number of these adsorbents target specific marine microorganisms and cannot overcome the serious hazards of diverse marine biological entities.^[^ ^18^ ^]^ Without specific targets for antimicrobial activity, ROS are reported to kill biological entities by acting on the ubiquitous organic components of biological cells and thus have a high potential to harbor broad anti‐biofouling activity.^[^ ^11b^ ^]^

**Scheme 1:**
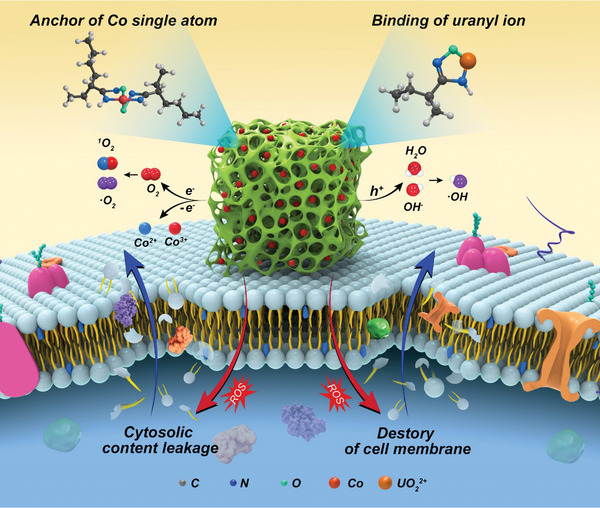
Schematic diagram for antimicrobial and uranium adsorption mechanisms of polyacrylamidoxime‐cobalt (PAO‐Co) adsorbent.

The amidoxime group is the most widely studied functional group for extracting uranium from seawater because of its relatively high selectivity and binding affinity for uranyl ions.^[^ ^19^ ^]^ However, the amidoxime group has also been reported to bind to other divalent metal cations, including Co^2+^.^[^ ^20^ ^]^ Thus, the anchoring of divalent metal ions by the amidoxime group to synthesize single atoms with ROS‐producing ability would be a potential strategy to fabricate uranium adsorbents with anti‐biofouling activity. In this study, the polyacrylamidoxime (PAO) uranium adsorbent was used to construct the amidoxime group‐anchored cobalt (Co) single‐atom material (PAO‐Co) for anti‐biofouling activity (**Scheme** **1**). The pre‐loaded cobalt ion (Co^2+^) bound by the amidoxime group was transferred into Co single atom by ball milling. Neighboring nitrogen (N) and oxygen (O) atoms from the amidoxime group stabilized the Co single atoms. Active Co single atoms promoted the formation of ROS, which destroyed marine fouling biological entities, including marine bacteria and marine algae, with high inhibition activity. In natural seawater, the adsorbent demonstrated a high adsorption capacity of 9.7 mg g^−1^, which was among the best‐performing uranium adsorbents. This study is the first to report the application of single atoms for controlling biofouling during uranium extraction from seawater. The findings of this study provide a novel strategy for fabricating anti‐biofouling uranium adsorbents and will inspire the design of novel materials with antimicrobial activity.

## 2 Results and Discussion

### 2.1 Synthesis and Characterization of PAO‐Co

**Figure 1:**
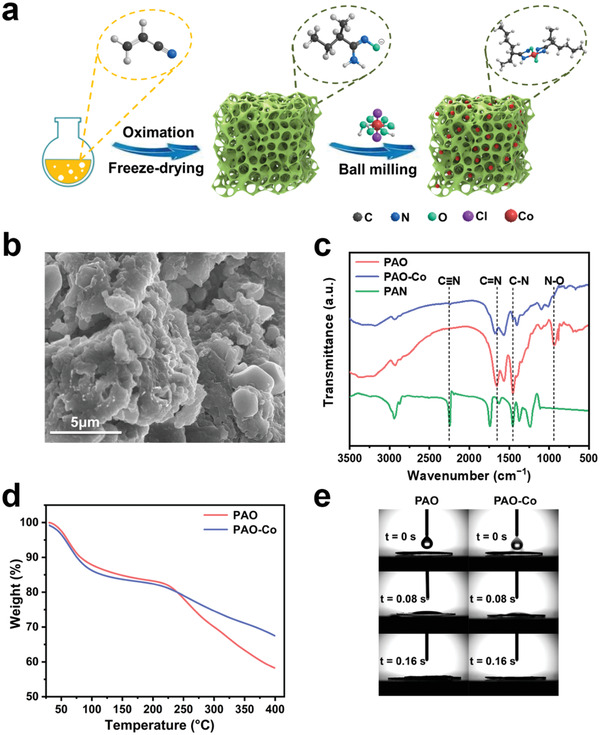
Synthesis and characterization of the material PAO‐Co. a) Schematic diagram for the preparation of Co‐single‐atom anchored uranium adsorbent PAO‐Co. b) SEM images of PAO‐Co. c) FTIR spectra of PAN, PAO, and PAO‐Co. d) Thermal stability of PAO and PAO‐Co. e) Hydrophilicity of dried PAO and PAO‐Co.

The material used in this study was fabricated by preloading Co^2+^ into PAO, and Co^2+^ was transformed into Co single atoms by ball‐milling (**Figure** **1**a). The mass ratio of PAO and CoCl_2_ used for preparing the material was optimized to screen materials with high antimicrobial activity and uranium adsorption capacity. The result shows that PAO‐Co exhibits relatively high antimicrobial activity and uranium adsorption ability when the masses of PAO and CoCl_2_ are 500 and 100 mg, respectively, (Table S1, Supporting Information). An increase in the mass ratio caused a significant decrease in antimicrobial activity, and a decrease in the mass ratio led to a significant loss of uranium adsorption efficiency. Thus, this mass ratio was selected for material fabrication by ball‐milling. During the ball‐milling process, high‐energy collisions of the balls strengthened the interaction between PAO and Co^2+^, forming chemical structural defects. These defects further act as effective recombination centers for electron‐hole pairs and provide excited electrons to transform Co^2+^ into Co single atoms.^[^ ^21^ ^]^ The prepared PAO‐Co existed as an aerogel with a rough microcosmic surface (Figure 1b). Fourier transform infrared analysis revealed that the nitrile group in the original polyacrylonitrile (PAN) was transformed into the amidoxime group after the amidoximation process, which was confirmed by the weakening of the peak intensity of the C≡N group (2251 cm^−1^) and appearance of the peaks for the C=N group (1655 cm^−1^), C—N group (1411 cm^−1^), and N—O group (935 cm^−1^) (Figure 1c). Furthermore, after the addition of the Co element, the peak intensities for C=N, C—N, and N—O, decreased significantly in PAO‐Co after adding Co, indicating that Co could be bound by N and O atoms in the material. The thermal tolerance analysis of the material was also determined, demonstrating rapid weight loss for PAO at temperatures higher than 230 °C (Figure 1d). However, this result was not observed for PAO‐Co. The increase in the temperature stability of PAO‐Co was attributed to the interaction of Co with the amidoxime groups, stabilizing the structure of the material. Compared with PAO, PAO‐Co shows higher hydrophilicity due to the existing non‐transformed Co^2+^ interacting with water to increase hydrophilicity (Figure 1e), which can benefit the application of this material in seawater.

### 2.2 Confirmation of the Co Single Atoms in PAO‐Co

**Figure 2:**
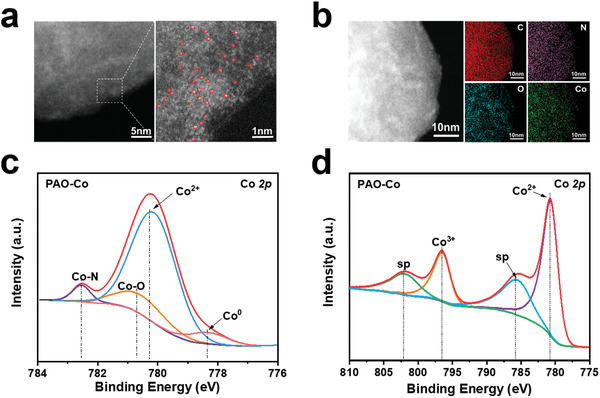
Characterization of the single atom in PAO‐Co. a) HAADF‐STEM image of PAO‐Co. The isolated bright dots marked with light‐red circles represent the Co single atoms. b) TEM image along with EDS mapping of PAO‐Co. c) Co 2*p* XPS spectra of PAO‐Co before use in the light‐induced anti‐biofouling activity assay. d) Co 2*p* XPS spectra of PAO‐Co after use in the light‐induced anti‐biofouling activity assay.

Aberration‐corrected high‐angle annular dark‐field scanning transmission electron microscopy (HAADF‐STEM) was used to analyze the fabricated material to determine the presence of Co single atoms and clarify the atomic structure of the single atom in PAO‐Co (**Figure** **2**a). Several bright spots were observed in the image of PAO‐Co, which were attributed to the presence of Co single atoms or Co clusters.^[^ ^22^ ^]^ Energy dispersive spectroscopy (EDS) analysis of the material also confirmed the uniform distribution of Co and other original elements, including C, N, and O, which further proved that the Co atoms were separately dispersed in the material (Figure 2b). X‐ray photoelectron spectroscopy (XPS) further analyzed the presence of Co atoms in PAO‐Co. The XPS spectra of PAO‐Co also confirmed the successful combination of Co atoms, which were absent in PAO (Figure S1, Supporting Information). The separation of the peaks for the Co element shows that Co exists in PAO‐Co in the form of Co—N (782.5 eV), Co—O (780.5 eV), Co^2+^ (780.1 eV), and Co^0^ (778.4 eV) (Figure 2c).^[^ ^23^ ^]^ Based on inductively coupled plasma mass spectrometry (ICP‐MS) and XPS analyses, the mass ratio of the total Co element and Co single atoms comprise of 8.49% and 0.71% of the material, respectively. The interaction between Co with N and O, which belongs to the amidoxime group, proves that Co element is bound by the amidoxime group. The state of Co element after use in the light‐induced anti‐biofouling activity assay was also determined. The result of the XPS analysis revealed that the zero‐valent Co element was transformed into the valent of Co^2+^ and Co^3+^, represented by the two satellite peaks at 785.5 and 801.9 eV, respectively (Figure 2d).^[^ ^23^, ^24^ ^]^ After using the material for antimicrobial activity assay, the total amount of the Co element is also reduced from 84.9 mg g^−1^ in the initial material to 41.5 mg g^−1^ in the used material (Figure S2, Supporting Information). The Co content decreases because the material cannot completely bind the Co ions owing to the higher binding affinity of the amidoxime group to the uranyl ion than to the Co ion.

### 2.3 Antimicrobial Properties and Antimicrobial Mechanism

**Figure 3:**
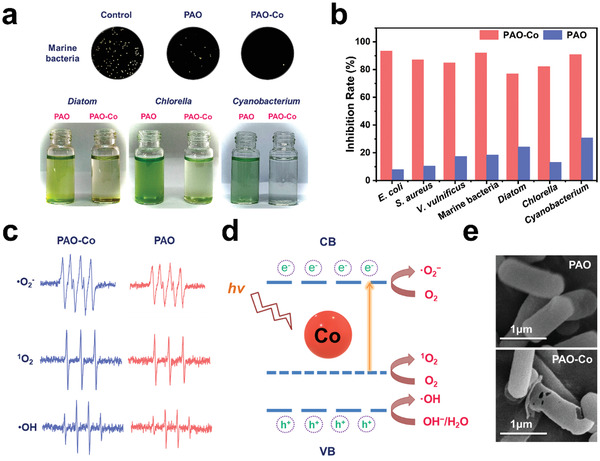
Antimicrobial activity and mechanism of PAO‐Co. a) Antimicrobial activity against the tested marine bacteria and algae. b) Inhibition ability against the growth of the microorganisms. c) Detection of the ROS produced by PAO‐Co under light irradiation. d) Schematic diagram for the production mechanism of ROS. e) SEM images of PAO and PAO‐Co treated *E. coli* cells.

Considering the potential antimicrobial activity of Co single atoms anchored material PAO‐Co, its antimicrobial activity was first tested against marine bacteria and algae under natural light irradiation. The results showed that PAO did not significantly inhibit the growth of marine microorganisms (**Figure** **3**a,b). In contrast, Co single atoms anchored material PAO‐Co significantly inhibited the growth of a broad range of microbial species with inhibition rates ranging from 77.0% to 93.4%, confirming the antimicrobial activity of the fabricated material. Considering the low concentration of Co single atoms in PAO‐Co, the Co single atom in the fabricated material possessed high efficiency in controlling microorganisms. The production of ROS was analyzed by electron spin resonance (ESR) to elucidate the antimicrobial mechanism of PAO‐Co. The results indicated that PAO‐Co produced more ROS than PAO with the irradiation of light, including the production of ^1^O_2_, ·OH, and·O_2_^−^, which was attributed to the presence of the Co single atoms in PAO‐Co (Figure 3c). The anchored Co single atoms decreased the band gap of PAO‐Co from 6.70 eV of PAO and to 3.50 eV, which significantly reduced the energy barrier and facilitated the generation of excited electrons (Figures S3 and S4, Supporting Information).^[^ ^25^ ^]^ According to previous reports, single metal atoms possess a high ability to trap elections and absorb oxygen because of their high chemical activity. The Co single atom in PAO‐Co acts as electron acceptor to promote the transfer of electrons in PAO and improve the separation efficiency of the electron‐hole pairs. The electron received by the Co single atom is further transferred to O_2_ adsorbed by Co single atom to produce ROS, including ·O_2_^−^ and ^1^O_2_ (Figure 3d).^[^ ^21c^ ^]^ Simultaneously, H_2_O and OH^−^ is oxidized by the residual electron holes to form ·OH. These ROS further catalyzed the degradation of organic components in the microbial cell, leading to the death of microorganisms.^[^ ^24^, ^26^ ^]^ The observation of the *Escherichia coli* cells treated with PAO and PAO‐Co revealed that the PAO‐Co treated bacterial cells were destroyed. However, the bacterial cells treated with PAO maintained an integrated cell structure, which confirmed the antifouling mechanism of PAO‐Co (Figure 3e).

### 2.4 Uranium Adsorption Performance

**Figure 4:**
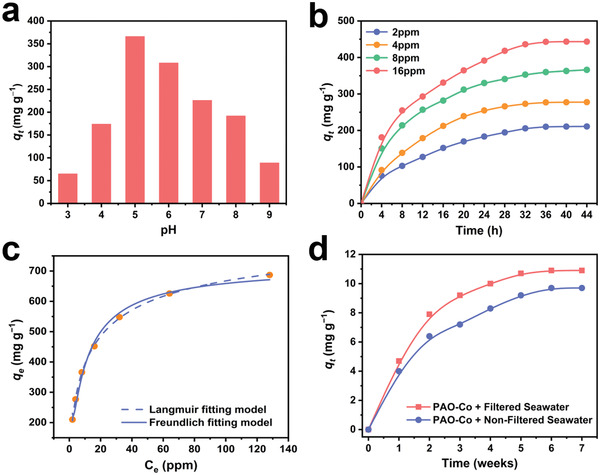
Uranium adsorption performance. a) Optimal pH for uranium adsorption. b) Adsorption kinetics in uranium‐spiked simulated seawater. c) Adsorption isotherm of PAO‐Co. d) Extraction of uranium from natural seawater with and without removing marine biofouling.

The uranium adsorption ability of PAO‐Co was first determined in uranium‐spiked simulated seawater. Optimal pH analysis for uranium adsorption showed that PAO‐Co achieved the highest uranium adsorption capacity of 366 mg g^−1^ in uranium‐spiked simulated seawater with a concentration of 8 ppm at pH 5.0 (**Figure** **4**a), which was consistent with that of the other amidoxime group‐based adsorbents.^[^ ^16^, ^27^ ^]^ Furthermore, the adsorption kinetics in uranium‐spiked simulated seawater shows that the adsorbent achieved a high uranium adsorption capacity of 443 mg g^−1^ in uranium‐spiked simulated seawater with a concentration of 16 ppm and an equilibrium adsorption time of 34 h (Figure 4b). Compared with PAO, the uranium adsorption capacity of PAO‐Co is decreased, due to the occupied amidoxime group by the Co single atom.^[^ ^28^ ^]^ Based on the Co atom content in PAO‐Co, approximately 13.6% of the functional amidoxime groups were occupied by Co atoms. Accordingly, the uranium adsorption capacity of PAO‐Co was approximately 918 mg g^−1^. The equilibrium adsorption isotherm showed that PAO‐Co possessed the highest uranium adsorption capacity of 687 mg g^−1^ in uranium solution with a concentration of 128 ppm, which was near to the theoretical highest uranium adsorption capacity (Figure 4c). The adsorption isotherm fitted well with the Langmuir model, indicating that the adsorbent bound uranium mainly via chemical adsorption. The analysis of the uranium‐loaded PAO‐Co by EDS revealed that uranium was adsorbed by the adsorbent (Figure S5, Supporting Information). Additionally, the analysis of uranium‐loaded PAO‐Co by XPS showed that the valence state of uranium did not change after its adsorption under irradiation, indicating that there was no reduction of uranium (Figure S6, Supporting Information). The reusability was one of the key factors that influence the economic cost of the adsorbent for practical uranium extraction. More than 91% of bound uranium can be eluted with an elution solution containing 1 m Na_2_CO_3_ and 0.1 m H_2_O_2_. The desorbed material can be further ball milled with Co^2+^ to regenerate the material. The results showed that, after being regenerated for 4 times, the material remains more than 70% of the initial uranium adsorption capacity and 90% of the initial antibacterial activity, respectively (Figure S7, Supporting Information).

The uranium extraction capacity of the adsorbent was tested in natural seawater under light irradiation. A high uranium extraction capacity of 9.7 mg g^−1^ was achieved after soaking the adsorbent in natural seawater for 49 days and the material still maintained integrated structure. Compared with the uranium extraction performance in unfiltered natural seawater of the other amidoxime group‐based adsorbents with antibiofouling activity, this material is one of the best adsorbents for uranium extraction (Table S2, Supporting Information). Natural seawater was filtered through a 0.22‐µm sterile filter to remove marine biological entities, including marine bacteria and algae, to determine the influence of marine biofouling on uranium extraction. The results demonstrated that the existence of marine biological entities resulted in only 11% attenuation of the uranium adsorption capacity (Figure 4d). In comparison, marine biofouling was reported to lead to 30–50% attenuation of the uranium extraction capacity of other adsorbents without anti‐biofouling activity.^[^ ^29^ ^]^ Considering the ability of PAO‐Co to inhibit the growth of marine microorganisms and destroy bacterial cells, PAO‐Co possesses a promising anti‐biofouling ability. Compared with other strategies used for controlling biofouling during uranium extraction from natural seawater, this method demonstrated a broad antimicrobial spectrum, comparable inhibition rates against marine microorganisms, and less interference of biofouling on uranium adsorption capacity.^[^ ^30^ ^]^ This result indicates that the PAO‐Co material is highly resistant to the influence of marine biofouling.

## 3 Conclusion

In conclusion, based on the redox activity of the single atom and the function of the amidoxime group in anchoring Co^2+^ ions, the Co single‐atom anchored material, designated as PAO‐Co, was fabricated for anti‐biofouling during uranium extraction from natural seawater. The material exhibited excellent and broad inhibition of the growth of marine biological entities with the highest inhibition rate of 93.4%. This antimicrobial ability is attributed to the production of ROS by Co single atoms under natural light irradiation. The adsorbent realizes a high uranium extraction capacity of 9.7 mg g^−1^ in natural seawater owing to the antimicrobial activity of PAO‐Co. This uranium extraction capacity is lower than the adsorbent soaking in seawater without marine biological entities by only 11%, suggesting the high resistance of the adsorbent to the influence of marine biofouling on the uranium extraction ability. Single atoms are used for anti‐biofouling during uranium extraction from natural seawater for the first time, thereby providing a new direction for the preparation of adsorbents for marine fouling prevention and control.

## 4 Experimental Section

### Preparation of PAO‐Co

To synthesize PAO, NH_2_OH·HCl was completely dissolved in dimethylformamide (DMF) at a ratio of 5.6% (m V^−1^). Then, NaOH and Na_2_CO_3_ were added to adjust the pH of the solution to neutral. After vigorous stirring for 1 h at 45 °C, PAN was added to the mixture slowly at a ratio of 8% (m V^−1^), and the temperature was increased to 65 °C for an additional 24 h. Furthermore, an additional amidoxime process was performed to complete the amidoximation of PAN by further adding NH_2_OH·HCl. NH_2_OH·HCl was completely dissolved in DMF at a ratio of 9.3% (m V^−1^), and NaOH and Na_2_CO_3_ were used to adjust the pH of the solution to neutral. After stirring for 10 min, this solution was added to the prepared mixture of PAN, and the reaction was conducted at 65 °C for another 24 h. After cooling to room temperature, the mixture was centrifuged at 10 000 rpm for 10 min to collect amidoximated PAN.^[^ ^31^ ^]^ The supernatant was collected and washed three times with anhydrous ethanol to remove residual DMF. The alcohol‐washed solution was then filtered by suction filtration and dried in an oven at 40 °C to obtain PAO powder.^[^ ^32^ ^]^

The PAO powder was dissolved in NaOH solution (0.3 m) and allowed to stand for 2 h to prepare PAO‐Co. The solution was then freeze‐dried for 48 h at −50 °C to form the PAO aerogel (Figure S8, Supporting Information).^[^ ^30^, ^33^ ^]^ CoCl_2_·6H_2_O was calcined at 130 °C for 4 h to remove the bound water.^[^ ^34^ ^]^ Anhydrous cobalt chloride (100 mg) was mixed with the PAO aerogel (500 mg) and ground in a planetary ball mill at 800 rpm for 36 h to fabricate PAO‐Co material.^[^ ^21^, ^35^ ^]^

### Material Characterization

The samples were observed through HAADF‐STEM (JEM‐ARM200F, JEOL Ltd., Japan) to obtain atomic‐resolution images of the material. The morphology and composition of the adsorbent were analyzed by SEM (GeminiSEM 300, Carl Zeiss AG, Germany) EDS (JEM‐ARM200F, JEOL Ltd., Japan). The valence states of the atoms in the sample were analyzed through XPS (Axis Supra, Kratos Analytical Ltd., UK) using a monochromatic Al X‐ray source (1486.6 eV) on a 5000C system. Paramagnetic substances with uncoupled electrons were detected through ESR (A300‐10/12, Bruker, Germany). Uranium was quantitatively analyzed through the UV–vis spectrophotometry (UV–vis, LAMBDA 750, PerkinElmer, USA) and ICP‐MS (Thermo Scientific iCAP RQ, ThermoFisher Scientific, USA).

### Determination of Antibacterial Activity

Strains of *E. coli*, *Staphylococcus aureus*, *Vibrio vulnificus*, and marine bacteria were selected as the indicator strains to determine the antibacterial activity of PAO‐Co. Exponentially growing bacterial strains were added to sterilized Luria‐Bertani broth at a volume ratio of 2%. Then the material was added to the cultures at a concentration of 1 mg mL^−1^. A bacterial culture without adding the adsorbent was used as a control. The culture was cultivated at 37 °C for 3 h with shaking at 180 rpm, and the viability of the bacterial culture was determined using the dilution plate counting method. The inhibition rate was calculated by counting the number of colonies that formed on the plates. The antibacterial rate was calculated using Equation (1).

(1) $$\mathrm{IR}=\frac{C_{i}-C_{a}}{C_{i}}\times 100\%$$

where *C*_a_ (CFU mL^−1^) and *C*_i_ (CFU mL^−1^) are the bacterial concentrations of the adsorbent‐treated and untreated bacterial cultures, respectively.

Strains of *Dictyosphaerium* sp., *Chlamydomonas reinhardtii*, and *Synechococcus elongatus* were used to determine the antimicrobial activity of PAO‐Co against marine algae. The algae, together with the material, were added to the sterilized BG11 medium at a volume ratio of 10% and a concentration of 1 mg mL^−1^ and cultured at 25 °C with shaking at 180 rpm. Algae samples were collected every 24 h, and the colonies were counted under a microscope with a blood cell counting plate. The inhibition rate of the algae was calculated using Equation (1).

### Determination of Optimum pH for Uranium Absorption

Simulated seawater containing NaCl (438.607 mm) and NaHCO_3_ (2.297 mm) was prepared to mimic the salinity of natural seawater to determine the optimum pH of the prepared adsorbent for uranium adsorption.^[^ ^36^ ^]^ Uranyl nitrate was dissolved in simulated seawater to a final concentration of 8 ppm, and the pH of the uranium‐spiked simulated seawater was adjusted with NaOH and HCl to a final pH of 3.0, 4.0, 5.0, 6.0, 7.0, 8.0, and 9.0. The dried adsorbent (5 mg) was added to pH‐adjusted uranium‐spiked simulated seawater (500 mL), and uranium adsorption was performed by shaking at 33 °C and 130 rpm. The samples were collected every 4 h until the adsorption reached equilibrium, and the uranium concentration was determined through UV–vis spectrophotometry. The solution with a known concentration of uranium was used to prepare a standard curve, and the uranium concentration in the sample was calculated. The amount of uranium absorbed by the adsorbent was calculated using Equation (2).

(2) $$q_{t}=\frac{\left(C_{0}-C_{t}\right)V}{m}$$

where *q*_t_ (mg g^−1^) is the uranium adsorption capacity after contact time *t*, *C*_0_ (mg L^−1^) is the initial uranium concentration, *C_t_* (mg L^−1^) is the uranium concentration at time *t*, *V* (L) is the volume of the uranium solution used, and *m* (g) is the weight of the used adsorbent.

### Adsorption Kinetics of Uranium in Simulated Seawater

To determine the adsorption kinetics of the adsorbent for uranium, uranium‐spiked simulated seawater was prepared (500 mL), and the optimized pH was adjusted for uranium adsorption. The adsorbent (5 mg) was added to the simulated seawater, and the adsorption process was performed by shaking at 33 °C and 130 rpm. The simulated seawater samples were collected every 4 h until adsorption equilibrium was reached. The uranium concentration in the solution was determined through UV–vis spectrophotometry. The uranium adsorption capacity was calculated using Equation (2).

### Isothermal Adsorption Curve

To analyze the main adsorption modes of the adsorbent, the adsorbent (5 mg) was added to uranium solutions (500 mL) of different uranium concentrations at pH of 6.0 to determine the uranium adsorption capacity.

The Langmuir adsorption isotherm was fitted with Equation (3).

(3) $$\frac{C_{e}}{q_{e}}=\frac{C_{e}}{q_{m}}\;+\frac{1}{k_{1}q_{m}}$$

where *q*_e_ (mg g^−1^) is the adsorption capacity of uranium at equilibrium, *c*_e_ (mg L^−1^) is the equilibrium concentration, *q*_m_ (mg g^−1^) is the maximum amount of adsorption, and *k*_1_ (L mg^−1^) is the equilibrium constant related to the binding strength. The Freundlich adsorption isotherm was fitted using Equation (4).

(4) $$q_{e}=k_{2}C_{e}^{\frac{1}{n}}$$

where *q_e_* (mg g^−1^) is the uranium adsorption capacity at equilibrium, *c*_e_ (mg L^−1^) is the equilibrium concentration of uranium, *k*_2_ (L g^−1^) is an equilibrium constant related to the binding strength, and *n* is the equilibrium constant.

### Uranium Adsorption in Natural Seawater

Natural seawater collected from the South China Sea near Wanning City, Hainan Province, China, was used without adding uranium to determine the uranium extraction capacity of the adsorbent in natural seawater. The natural seawater was also filtered through a 0.22‐µm filter to remove marine biological entities and suspension particle contaminants. For the test, adsorbent (10 mg) was added to natural seawater (100 L) and allowed to soak for 56 days. The concentration of the loaded uranium was determined using ICP‐MS every week.^[^ ^37^ ^]^

## Conflict of Interest

The authors declare no conflict of interest.

## Supporting information

Supporting Information

## Data Availability

The data that support the findings of this study are available from the corresponding author upon reasonable request.
