# Supplementary material for: Amidoxime Group‐Anchored Single Cobalt Atoms for Anti‐Biofouling during Uranium Extraction from Seawater
Source: Adv Sci (Weinh). 2022 Jan 22;9(10):2105008. doi: 10.1002/advs.202105008 (PMC8981433; doi:10.1002/advs.202105008)
Supplement: Supplementary file 1 — Supporting Information [file ADVS-9-2105008-s001.pdf]

ADVANCED SCIENCE
Open Access

Supporting Information

for *Adv. Sci.*, DOI 10.1002/advs.202105008

Amidoxime Group-Anchored Single Cobalt Atoms for Anti-Biofouling during Uranium Extraction from Seawater

Wenyan Sun, Lijuan Feng, Jiacheng Zhang, Ke Lin, Hui Wang, Bingjie Yan, Tiantian Feng, Meng Cao, Tao Liu, Yihui Yuan* and Ning Wang**

ADVANCED SCIENCE
Open Access

Supporting Information

for *Adv. Sci.,* DOI: 10.1002/advs.202105008

Amidoxime group-anchored single cobalt atoms for antibiofouling during uranium extraction from seawater

Wenyan Sun, Lijuan Feng, Jiacheng Zhang, Ke Lin, Hui Wang, Bingjie Yan, Tiantian Feng, Meng Cao, Tao Liu, Yihui Yuan*, and Ning Wang**

Supporting Information

Amidoxime group-anchored single cobalt atoms for anti-biofouling during uranium extraction from seawater

Wenyan Sun, Lijuan Feng, Jiacheng Zhang, Ke Lin, Hui Wang, Bingjie Yan, Tiantian Feng, Meng Cao, Tao Liu, Yihui Yuan*, and Ning Wang**

W. Sun, L. Feng, J. Zhang, K. Lin, Dr. H. Wang, B. Yan, T. Feng, M. Cao, Prof. T. Liu, Prof. Y. Yuan, and Prof. N. Wang

State Key Laboratory of Marine Resource Utilization in South China Sea, Hainan University

Haikou 570228, P. R. China

Email: wangn02@foxmail.com, yuanyh@hainanu.edu.cn, huiw0318@163.com

Supporting Figures

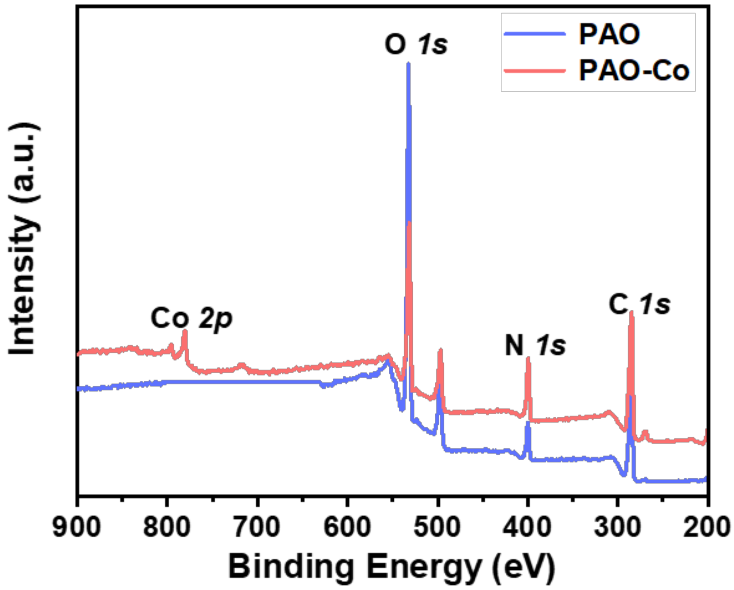

Figure S1. XPS spectra of PAO and PAO-Co.

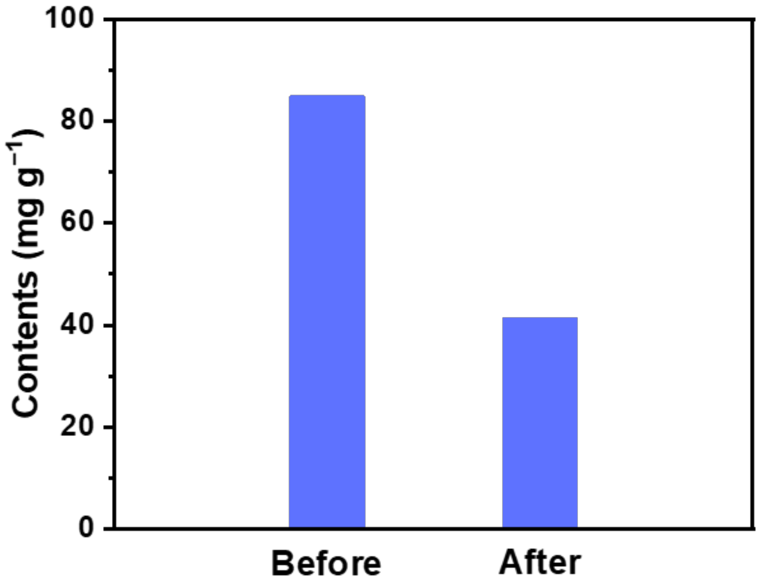

Figure S2. Contents of Co element in PAO-Co before and after being used for the light induced antibiofouling ability assay.

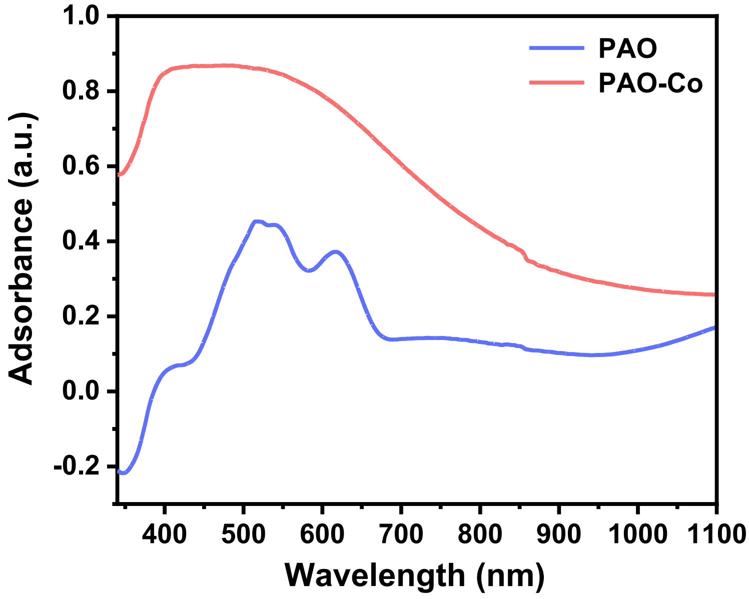

Figure S3. UV-visible-near infrared adsorption spectra of PAO and PAO-Co.

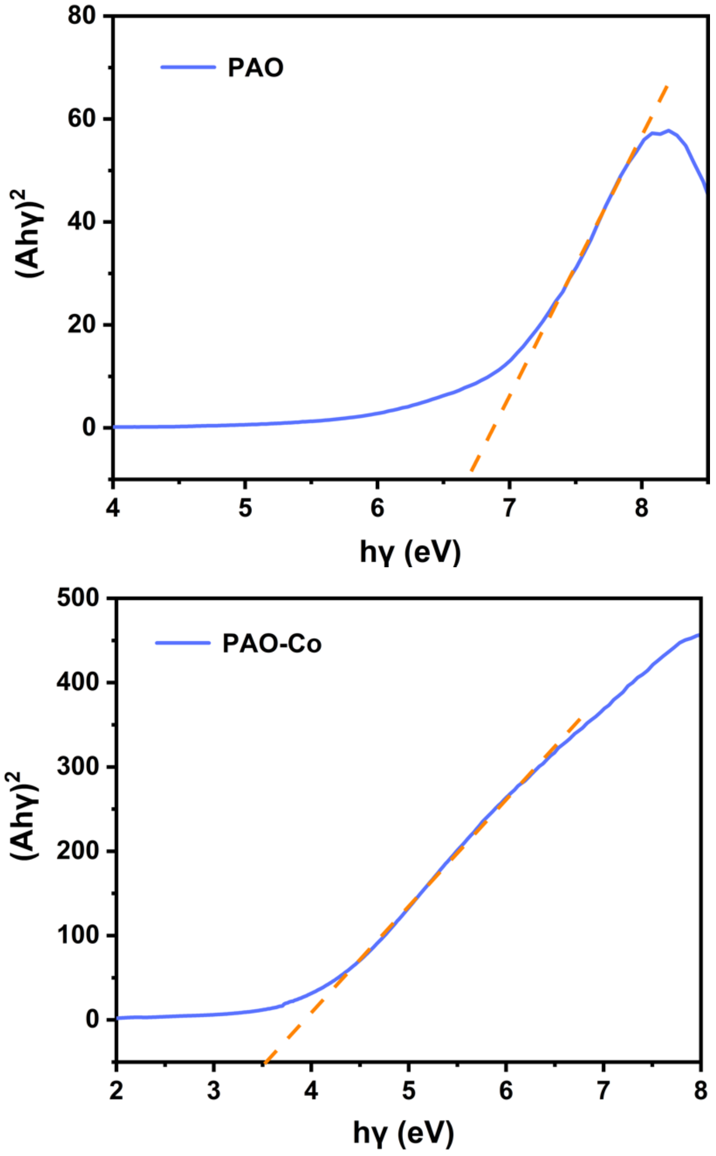

Figure S4. Band gap of PAO and PAO-Co.

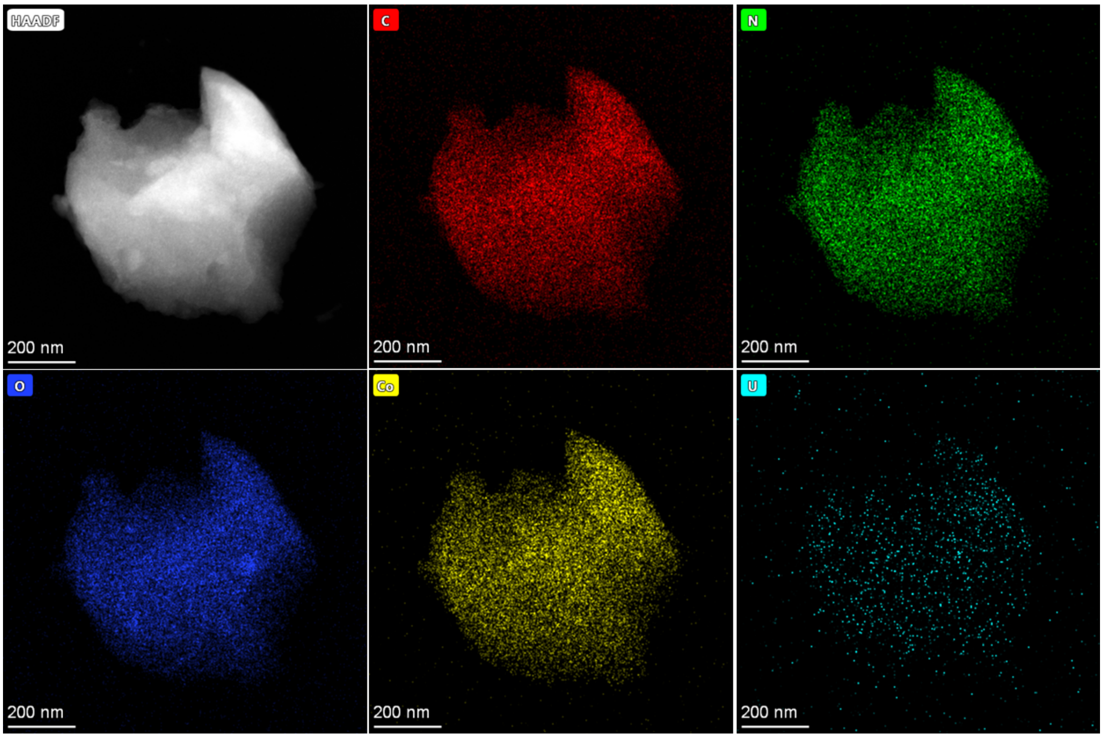

Figure S5. EDS mapping of elements on uranium loaded PAO−Co.

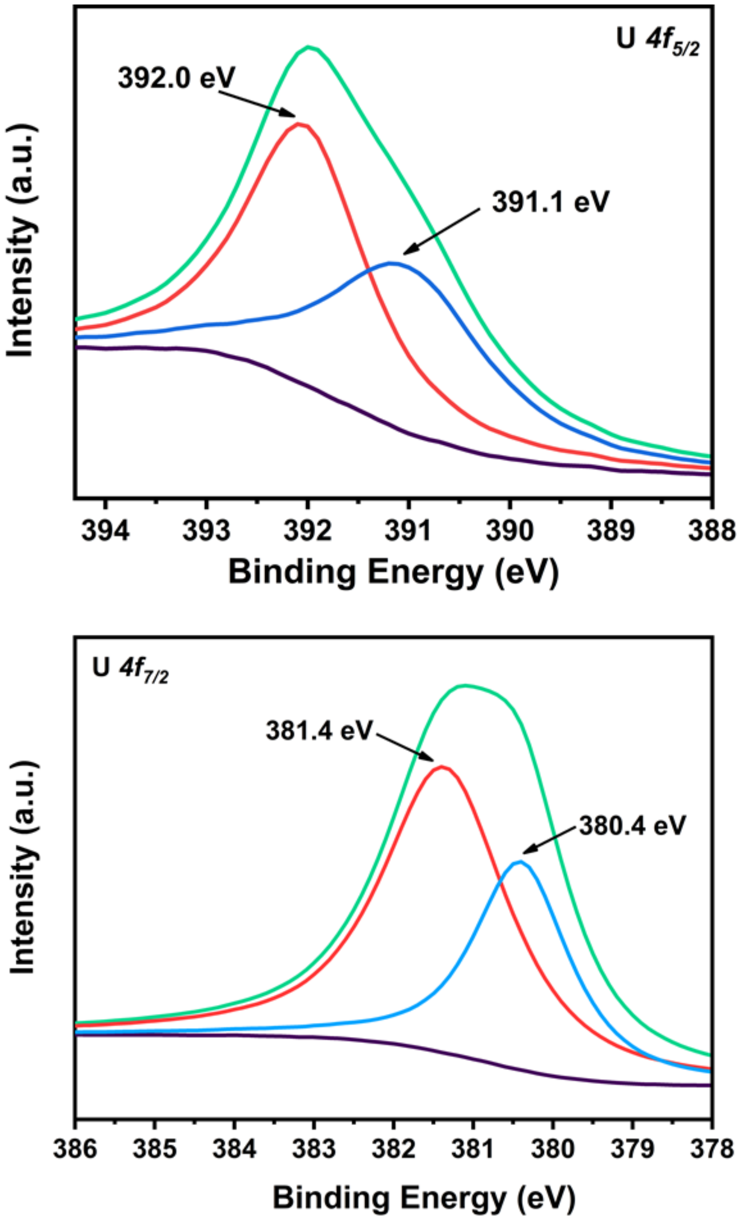

Figure S6. High-resolution XPS spectrum of uranium adsorbed in PAO−Co under light irradiation.

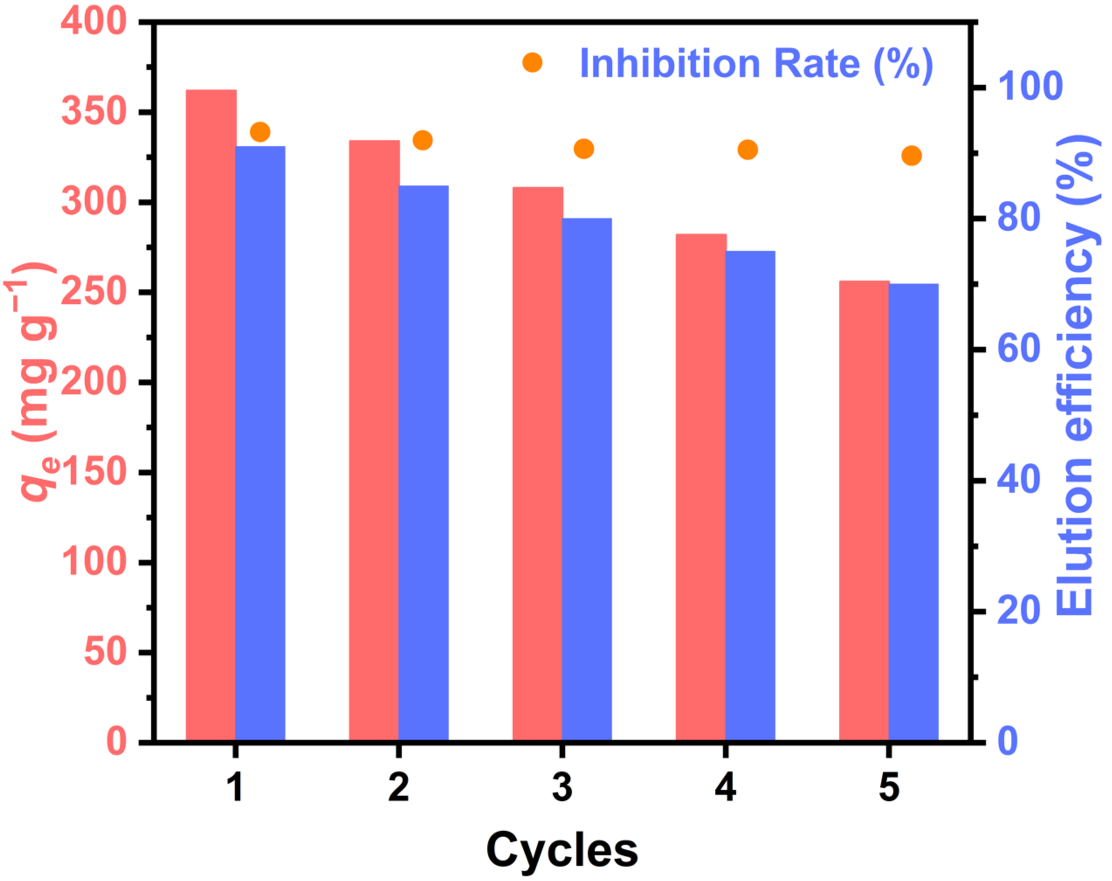

Figure S7. Uranium adsorption and antibacterial properties of regenerated materials.

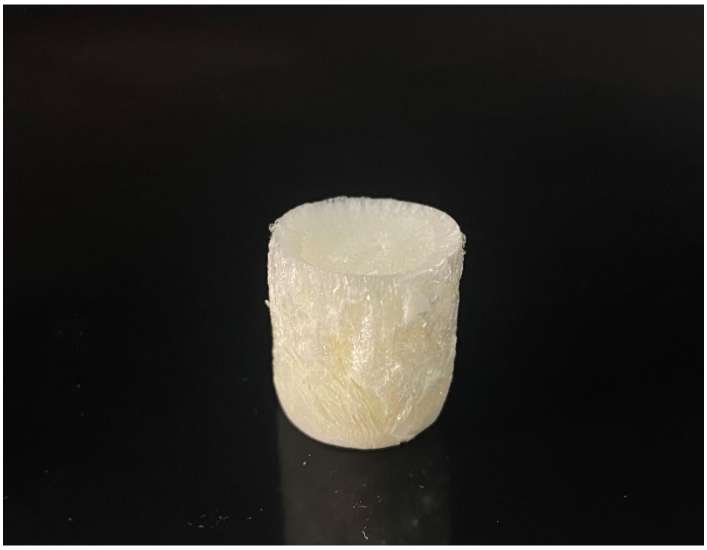

Figure S8. Macroscopic morphology of the PAO aerogel.

Supporting Tables

Table S1. Optimization of the mass of PAO and $CoCl_2$ for fabricating the material. The uranium adsorption capacity is determined in 8 ppm uranium-spiked simulated seawater. The antimicrobial activity is tested by using strain of *E. coli* as indicator strain.

$PAO/CoCl_2$ (mg / mg)	Inhibition rate (%)	Uranium extraction capacity (mg g^{-1})
500 / 60	74.6	423
500 / 80	77.3	390
500 / 100	93.4	366
500 / 120	94.1	286
500 / 140	94.5	223

Table S2. Uranium extraction performance of the amidoxime group-based adsorbents with antibiofouling activity.

Adsorbent	Uranium extraction (mg g^{-1})	Time (d)	Reference
Zn@AO fiber	0.3	30	[1]
AO-HNTs	9.01	30	[2]
DC-PAO	6.42	7	[3]
PP-g-AO_3	5.22	49	[4]
PAO PNMs	9.35	35	[5]
NDA-TN-AO	6.06	27	[6]
Fe_3O_4@TiO_2-AO	0.0875	33	[7]
COF-HHTF-AO	5.12	25	[8]
PAO-PHMB-A	3.19	30	[9]
NC-PAO DN	8.62	25	[10]
PA-PAO/CS NFs	4.91	10	[11]
PAO-CB	8.59	56	[12]
Anti-PAO	9.29	30	[13]
Zn^{2+}-PAO	9.23	28	[14]
3D hierarchical porous amidoxime fibers	11.5	90	[15]
PAO hydrogel	4.87	28	[16]
PAO-Co	9.70	42	This work

Reference

[1] J. Ao, Y. Yuan, X. Xu, L. Xu, Z. Xing, R. Li, G.-Z. Wu, X. Guo, H. Ma, Q. Li, *Ind. Eng. Chem. Res.* **2019**, *58*.

[2] S. Zhao, Y. Yuan, Q. Yu, B. Niu, J. Liao, Z. Guo, N. Wang, *Angew. Chem. Int. Ed.* **2019**, *58*, 14979.

[3] N. Wang, X. Zhao, J. Wang, B. Yan, S. Wen, J. Zhang, K. Lin, H. Wang, T. Liu, Z. Liu, C. Ma, J. Li, Y. Yuan, *Adv. Sci.* **2021**, 2102250.

[4] H. Zhang, L. Zhang, X. Han, L. Kuang, D. Hua, *Ind. Eng. Chem. Res.* **2018**, *57*.

[5] S. Shi, Y. Qian, P. Mei, Y. Yuan, N. Jia, M. Dong, J. Fan, Z. Guo, N. Wang, *Nano Energy* **2020**, *71*, 104629.

[6] W.-R. Cui, F.-F. Li, R.-H. Xu, C.-R. Zhang, X.-R. Chen, R.-H. Yan, R.-P. Liang, J.-D. Qiu, *Angew. Chem. Int. Ed.* **2020**, *59*, 17684.

[7] N. Li, P. Gao, H. Chen, F. Li, Z. Wang, *Chemosphere* **2022**, *287*, 132137.

[8] G. Cheng, A. Zhang, Z. Zhao, Z. Chai, B. Hu, B. Han, Y. Ai, X. Wang, *Sci. Bull.* **2021**, *66*, 1994.

[9] N. He, H. Li, L. Li, C. Cheng, X. Lu, J. Wen, X. Wang, *J. Hazard. Mater.* **2021**, *416*, 126192.

[10] R. Liu, S. Wen, Y. Sun, B. Yan, J. Wang, L. Chen, S. Peng, C. Ma, X. Cao, C. Ma, G. Duan, S. Shi, Y. Yuan, N. Wang, *Chem. Eng. J.* **2021**, *422*, 130060.

[11] D. Wang, Z. Liu, Y. Yue, X. Xu, D. Cai, C. Han, J. Song, J. Xiao, H. Wu, *Mater. Today Energy* **2021**, *21*, 100735.

[12] Y. Yuan, X. Guo, L. Feng, Q. Yu, K. Lin, T. Feng, B. Yan, K. V. Fedorovich, N.

Wang, *Chem. Eng. J.* **2021**, *421*, 127878.

[13] S. Shi, B. Li, Y. Qian, P. Mei, N. Wang, *Chem. Eng. J.* **2020**, *397*, 125337.

[14] B. Yan, C. Ma, J. Gao, Y. Yuan, N. Wang, *Adv. Mater.* **2020**, *32*, 1906615.

[15] X. Xu, H. Zhang, J. Ao, L. Xu, X. Liu, X. Guo, J. Li, L. Zhang, Q. Li, X. Zhao, B. Ye, D. Wang, F. Shen, H. Ma, *Energy Environ. Sci.* **2019**, *12*, 1979.

[16] C. Ma, J. Gao, D. Wang, Y. Yuan, J. Wen, B. Yan, S. Zhao, X. Zhao, Y. Sun, X. Wang, N. Wang, *Adv. Sci.* **2019**, *6*, 1900085.
